# Cost of a healthy and sustainable basic food basket in Brazil between 2009 and 2024

**DOI:** 10.11606/s1518-8787.2026060006878

**Published:** 2026-04-10

**Authors:** Eliseu Verly, Dirce Maria Lobo Marchioni, Semíramis Martins Álvares Domene, Isabela de Albuquerque Ribeiro, Flávia Mori Sarti

**Affiliations:** I Universidade do Estado do Rio de Janeiro. Instituto de Medicina Social. Departamento de Epidemiologia. Rio de Janeiro, RJ, Brasil; II Universidade de São Paulo. Faculdade de Saúde Pública. Departamento de Nutrição. São Paulo, SP, Brasil; III Universidade Federal de São Paulo. Instituto de Saúde e Sociedade. Departamento de Políticas Públicas e Saúde Coletiva. São Paulo, SP, Brasil; IV Universidade de São Paulo. Escola de Artes, Ciências e Humanidades. Gestão de Políticas Públicas. São Paulo, SP, Brasil

**Keywords:** Healthy Eating, Basic Food, Consumer Price Index, Cost Analysis

## Abstract

**OBJECTIVE:**

To evaluate the evolution of the cost of a proposed healthy and sustainable basic food basket, nationally and for metropolitan regions, between 2009 and 2024.

**METHODS:**

Food purchase data (quantities and amounts paid) from the 2008–2009 and 2017–2018 *Pesquisas de Orçamentos Familiares* (POF - Household Budget Surveys) were used. The food groups that make up the baskets were based on the Eat-Lancet planetary diet and official regulatory instruments*.* The Broad Consumer Price Index for foods and food groups was used to update the monthly prices of the items consumed by the Brazilian population in the context of the POFs that make up the proposed basic food basket. The accumulated inflation over the period was discounted to compare monetary values over time, calculating the real value of the basic food baskets.

**RESULTS:**

The real cost of the proposed national basic food basket remained relatively stable between 2009 and 2012, showing a subsequent upward trend until early 2017, including more significant growth between 2018 and 2024. A similar pattern was observed in the basic food baskets of the metropolitan regions. The cost (adult/month) represented on average 26% of the minimum wage in force in each year of the analysis period and 19% of the national average *per capita* income between 2010 and 2023. The share of food groups in the cost of the proposed basket remained stable over the period.

**CONCLUSION:**

There has been an increase in the real cost of the proposed healthy and sustainable basic food basket at national and regional level, particularly since 2018. Moreover, the value of the healthy and sustainable basic food basket showed an upward trend in relation to the average *per capita* income of the population, especially since 2018.

## INTRODUCTION

The first initiative to establish a basic food basket in Brazil was made by the decree-law that regulated the minimum wage (Decree-Law No. 399 of 1938), defined as the minimum wage necessary to meet the daily needs of “*food, housing, clothing, hygiene, and transportation*” of workers at a given time and in a given region of the country. The epidemiological scenario in the country was centered on the problem of hunger and nutritional deficiencies, particularly in terms of compromising the productivity of the workforce due to illness caused by malnutrition. The cost of the basic food basket stipulated at the end of the 1930s has been used for decades as an indicator of the cost of living and access to basic food for the population, as well as a parameter for estimating the minimum wage in the country, although the real value of the wage has lagged in various periods of history^1^.

Recently, a new composition of the basic food basket was defined by presidential decree, with the aim of subsidizing policies and programs to guarantee the human right to adequate food. The decree establishes compliance with the recommendations of the Guia Alimentar para a População Brasileira (Dietary Guidelines for the Brazilian Population) as one of the guidelines for the composition of the basic food basket, and the inclusion of ultra-processed foods is prohibited. The change in the composition of the basic food basket also seeks to encourage the promotion of sustainable food systems by considering the use of natural resources and greenhouse gas emissions involved in food production and marketing. The new basic food basket includes ten food groups, mostly made up of fresh or minimally processed foods. Other aspects of the food system, such as encouraging foods from socio-biodiversity and cultural acceptability, are covered by the decree and should be taken into account when proposing basic food baskets, i.e. when defining the types of food in each group and their respective quantities^2,3^.

Income and the price of food are barriers to access and acquisition of good quality food in sufficient quantity to maintain the health of the population^4^. The role of social determinants in the food-nutrition-health process has been highlighted in the literature worldwide, pointing to the relationship between socioeconomic conditions and food acquisition or consumption^5^. Empirical studies systematically point to an increase in the demand for healthy foods as a result of an increase in the population’s income or a reduction in the prices of food items associated with healthy eating patterns^6,7^. Thus, the cost of a healthy basic food basket is an important indicator for monitoring the population’s access to a minimum set of living conditions based on nutritional demands. Therefore, the aim of this study was to evaluate the evolution of the real and nominal cost of a healthy basic food basket in Brazil and its metropolitan regions over the last 15 years, between 2009 and 2024. In addition, the trajectory of the proportion of the family budget needed to purchase the basic food basket proposed in this study was estimated, as well as the evolution of the cost share of each food group in relation to the total cost of the basic food basket.

## METHODS

### Data Source

The database used to select the foods and their respective quantities was extracted from information on household food purchases, collected as part of the *Pesquisas de Orçamentos Familiares* (POF - Household Budget Surveys) conducted by the Instituto Brasileiro de Geografia e Estatística (IBGE - Brazilian Institute of Geography and Statistics) in 2008 and 2009. Food products purchased by household members during the data collection period were recorded in the collective purchase booklet in the context of the POF, including: quantity purchased, place, form of purchase and amount spent. As data collection took place over 12 months, the costs reported for each purchase item were adjusted to a single reference period (January 2009) using Extended National Consumer Price Index (IPCA). Information on sampling, data collection and other POF methodological procedures can be found on the survey website^8^ . Exclusively in the case of the Vitória metropolitan region, data from the 2017–2018 POF was used to coincide with periods in which inflation indices are available to update the region’s monetary values. The sampling and data collection procedures were similar for both editions of the POF.

### Defining the Composition of the Food Basket

Initially, the food groups used to make up the national and regional basic food baskets were defined. As theoretical references for the construction of the basic food basket proposal, federal government documents were considered, referring to Constitutional Amendment No. 132 (of December 20, 2023), Decree No. 11,936 (of March 5, 2024), and Ministry of Social Development and Fight against Hunger Ordinance No. 966 (of March 6, 2024). These documents adhere to the principles of healthy eating set out in the Dietary Guidelines for the Brazilian Population, defining the food groups in the basket and prohibiting the use of any ultra-processed foods. Next, when defining the quantities of each of the food groups, the main theoretical reference was the Eat-Lancet Reference Diet (planetary diet)^9^. This publication provides a range of recommended consumption values for the food groups for which there is sufficient evidence to reduce the risk of chronic non-communicable diseases. At this stage, some adaptations have been made:

- Given the cultural and nutritional importance of red meat, the maximum amount proposed by the planetary diet was defined, which is equivalent to approximately two 100 g portions per week (gross weight of 40 g per day, which is equivalent to 28 g after cooking). We chose to follow the ratio between beef and pork as reported by the Brazilian population, which led to an amount of beef above the limit set by the planetary diet. The implications of this change are addressed in the discussion section.
- Three 100 g portions of chicken and one 150 g portion of fish per week were defined (gross weight equivalent to 430 g and 215 g per week, respectively).
- Considering the cultural acceptance of beans, as well as their importance as a source of nutrients, an intermediate value was defined between the consumption observed in the population and the value optimized for nutritional adequacy^10,11^.
- The amount of oilseeds (such as chestnuts) was reduced to 5 g because they are not frequently present in the Brazilian diet and are expensive.
- Two portions a day of fruit and four portions of vegetables were defined, as well as one portion of tubers and starchy vegetables.
- The amount of cereals, which includes whole grains, processed cereals, and derivatives (bread and pasta) was calculated to complete the energy value of 2,000 kcal, with a limit of 60% of the total calories in the basket. 20 g was adopted for brown rice (equivalent to 50 g post-coction); this amount can be achieved by replacing 30% of the occasions on which white rice is consumed in the population, with a substantial estimated impact on deaths from chronic non-communicable diseases^10^.
- Coffee was included to represent the “Coffee, tea, *mate*, and spices” group because it is the most commonly reported item in this group. As there is no recommendation value for coffee consumption, the same average purchase quantity reported by each federation unit was adopted in the proposed basket.

Table shows the distribution of food groups according to the planetary diet proposal and this food basket proposal for Brazil. The composition of the basket was presented as quantities of food to be purchased, referred to as the “acquisition basket” (gross weights) and quantities to be actually consumed, referred to as the “consumption basket” (weights corrected for cooking and inedible portions).

**Table:** Reference quantities and proposed quantities of food groups to make up the national basic food basket (grossa and adjustedb quantities), Brazil.

	Eat-Lancet reference diet^a^	Basket (consumption^b^)	Basket (acquisition^c^)
Cereals	≤ 60%^d^	37%^d^	37%^d^
Brown rice		50	20
White rice		225	90
Bread		50	50
Noodles		23	10
Tubers and pasta vegetables	50 (0–100)	30	45
Vegetables	300 (200–600)	200	250
Fruits	200 (100–300)	210	300
Dairy products			
Milk	250 (0–500)	150	150
Cheese		20	20
Butter		5	5
Red meat	14 (0–28)		
Beef		24	34
Pork		4	6
Poultry	29 (0–58)	43	60
Fish	28 (0–100)	21	30
Eggs	13 (0–25)	20	20
Pulses	50 (0–100)	200	80
Peanuts, walnuts and chestnuts	50 (25–75)	5	5
Unsaturated oils	40 (20–80)	35	35
Added sugar	31 (0–31)	10	10
Coffee, tea, *mate*, and spices^e^	–	6	6

^a^ Reference diet and possible ranges (g/day).

^b^ Weight, in grams, removing inedible parts and adjusting for cooking factors.

^c^ Gross weight in grams (quantity to be purchased). Reference quantities refer to actual consumption quantities.

^d^ Share of total calories in the basket.

^e^ There is no consumption recommendation for this item.

We chose to include all the foods reported in each group, since they can all be part of the basket. The quantities proposed were proportional to the quantities purchased (*per capita*) by the families and added up to reach the quantities established for each food group. As an example, the average daily per capita purchase of bananas in the country was 16 g, which represents 28% of the total fruit purchased (59 g). In the composition of the basket, the banana’s share remains the same (28%), which is equivalent to 84 g out of the total fruit that should make up the basket (300 g). The calculation, however, was made for each state and the Federal District and the national average was calculated as the average of their quantities between states, weighted by the population size of each state. This procedure made it possible to consider the variability of the basket’s composition, giving more weight to food items that are consumed more frequently, thus respecting the cultural regionality of food.

### Food Basket by Metropolitan Region

This procedure was repeated for each of the 10 metropolitan regions considered by the IBGE when constructing the price indices. In terms of the sampling delimitation of the metropolitan regions, households were aggregated from the primary sampling units in each federation unit, belonging to the capitals and metropolitan regions, as described in the survey documentation. The metropolitan regions evaluated are: Belém, Fortaleza, Recife, Salvador, Vitória, Belo Horizonte, Rio de Janeiro, São Paulo, Curitiba, and Porto Alegre.

The quantities *per capita* per day of the food groups for the proposed healthy basic food baskets (Brazil and Metropolitan Regions) were adjusted to provide approximately 2,000 kcal/person/day.

### Monthly Cost of Food and Baskets

Changes in the price of each food item, measured by the *Sistema Nacional de Índice de Preços* (SNIPC - National System of Consumer Price Indexes) using the IPCA, were used to update the cost of the basic food basket. For the Brazilian basket, the IPCA estimated for Brazil was used, and for the metropolitan regions, their respective indices. Each item in each basket was paired with its respective SNIPC item. In the absence of a price index for a given item in a given period, the record for the food group was used. The reference price per kilogram for each food item was obtained from the collective expenditure module of the 2008–2009 POF (2017–2018 exclusively for the Vitória metropolitan region), whose reference period was January 2009 (January 2018 in the case of the 2017–2018 POF). The monetary update of the values of each food item *i* component of the basic food baskets for subsequent months (February 2009 to December 2024, or February 2018 to December 2024 for the Vitória metropolitan region) was based on the following equation:


$$p_{i}^{t+1}=p_{i}^{t}\times \left(1+\pi_{i}^{t,t+1}/100\right)$$

Where: $p^{t+1}{}_{i}=\text{ price of item }i$ in period *t+1*; $p_{i}^{t}=\text{ price of item }i$ in period *t*; $\pi_{i}^{t,t+1}=\text{ change in the price of item}i$ between *t* and *t+1* (IPCA for month *t+1* for each item).

Thus, the nominal value was calculated, i.e. the effective value for each period, for each item, and the total nominal cost of the basic food basket was calculated. For comparison purposes over time, it is necessary to discount the accumulated inflation over the period. Based on this procedure, called deflation, the real costs of the basic food baskets in the period were calculated. The following equation was used:


$$vr_{t}=\frac{vn_{t}}{IPCA_{t0-t}/IPCA_{t0-ref}}$$

Where: *vr*_t_ = cost of the basic food basket in month *t* discounted for inflation, *vn*_t_ = cost of the basic food basket actually practiced in month *t*, and *IPCA*_t0–t_ and *IPCA*_t0–ref_ = accumulated changes in the general inflation index (Brazil or metropolitan regions) between *t0* (January 2009 or January 2018) and month *t*, and between *t0* and the reference month (December 2024), respectively.

### Participation of Food Groups in the Cost of the Basket

For each month of the historical series, the share of each food group in the overall cost of each basic food basket was calculated to identify their respective weight in determining the population’s cost of living.

### Participation in the Family Budget

For each month in the historical series, the proportion of the average *per capita* household income used to purchase the basic food basket *per capita* was calculated. Estimates of average *per capita* income in Brazil were obtained from the continuous National Household Sampling Survey, whose historical series begins in 2012. In addition, the ratio between the monthly cost of basic food baskets for an adult individual and the value of the minimum wage in force in the respective period was calculated (expressed as a percentage). For these calculations, the nominal values of income, minimum wage, and cost of the baskets were used.

## RESULTS

**Figure 1:**
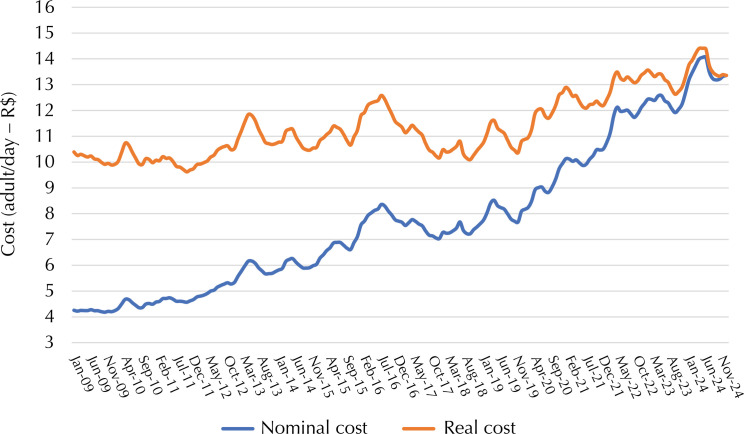
Real and nominal cost of the basic food basket (adult/day) in reais. Brazil, 2009 to 2024.

The *per capita* cost of the healthy and sustainable basic food basket in Brazil is shown in Figure 1. The nominal value for purchasing food starts at R$ 4.25 in January 2009 and reaches R$ 13.36 in December 2024. In real terms (adjusted for December 2024), the cost would be R$ 10.39 in January 2009. The real cost remained relatively stable until mid-2012, with an upward trend until early 2017. After a rapid period of reduction until early 2018, there was a significant upward trend, which continued throughout the following period. In November 2019, the real cost of the basic food basket was practically the same as in January 2018 (R$ 10.38 versus R$ 10.36). From this point on, there was a cumulative increase of 28% until December 2024.

**Figure 2:**
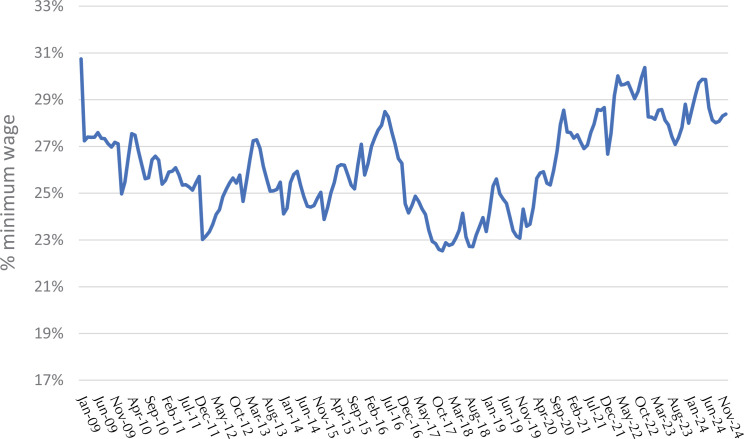
Percentage of the minimum wage needed to buy a healthy basic food basket. Brazil, 2009 to 2024.

**Figure 3:**
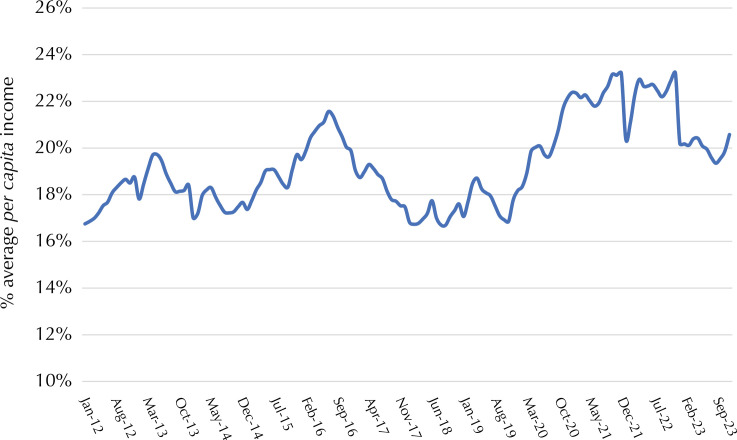
Percentage of average *per capita* income needed to buy a healthy basic food basket. Brazil, 2012 to 2023.

**Figure 4:**
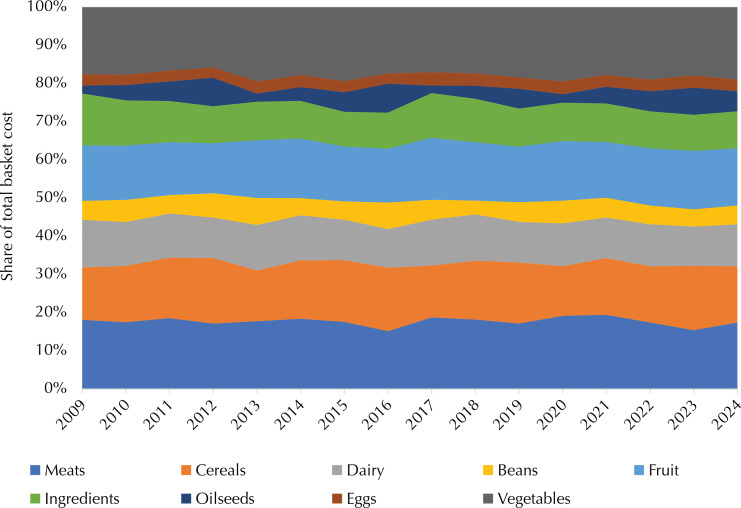
Relative share (%) of the costs of each food group in relation to the total cost of the basket. Brazil, 2009 to 2024.

The ratio between the monthly cost of a healthy basic food basket for an adult and the value of the minimum wage fell between 2009 and 2017, reaching a low of 23% in December 2017. An upward trend was observed from 2018 onwards, reaching a maximum value of 30% in November 2022 (Figure 2). The average over the period was 26%. The ratio between the value of the monthly basket per adult individual and *per capita* household income remained stable at around 18% until the end of 2019, with an upward trend in the following months and an apparent reduction from the beginning of 2023 (Figure 3). There was no significant change in the share of each food group in the total cost of the basket between 2009 and 2024. Fruits and vegetables accounted for about 30% of the total cost, followed by meats (17%), cereals (15%), dairy products (11%), and ingredients (oil, salt, sugar, olive oil), totaling about 10% (Figure 4).

**Figure 5:**
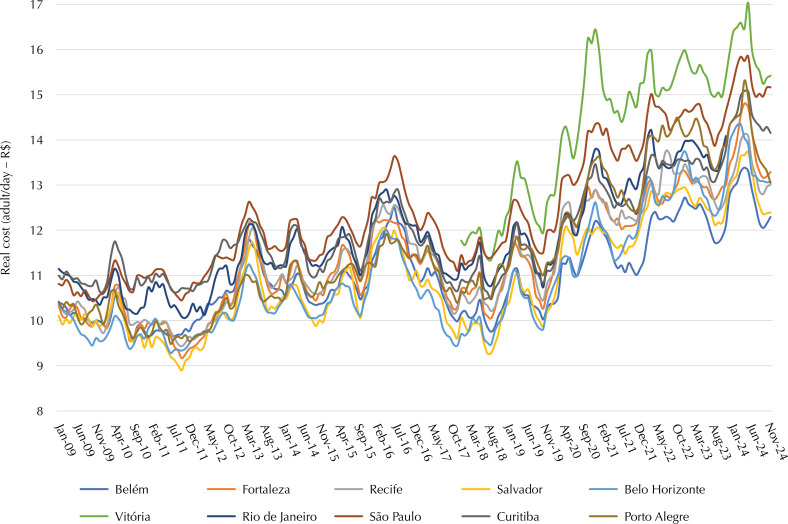
Real (deflated) cost of the basic food basket (adult/day) in Reais. Metropolitan regions, 2009 to 2024.

The evolution of the cost of regional baskets followed the same pattern as the national basket, with similar trajectories between metropolitan regions. The baskets that remained the least expensive for most of the period were in the metropolitan regions of Fortaleza, Belém, and Belo Horizonte. On the other hand, the most expensive baskets were mostly in the metropolitan regions of São Paulo and Rio de Janeiro (Figure 5).

## DISCUSSION

In this article, we evaluate the evolution of the cost of a proposed basic food basket for Brazil and its metropolitan regions, as well as its participation in the monthly family budget. Although the nominal cost is relevant because it represents the amount actually paid at a given time, its interpretation is limited because it carries the distorting effect of inflation. The isolated observation that costs increase over time is insufficient, since this variation is typically accompanied by adjustments in the population’s monetary income. On the other hand, the analysis of real costs removes the effect of inflation, making it possible to identify variations in purchasing capacity. Thus, based on the real costs of purchasing the items included in the proposed healthy basic food baskets, it is possible to identify a long period of stability (January 2009 to September 2018) followed by a sharp increase, accumulating 29% in the cost of the proposed basket over the period investigated. This increase varied from 15% to 30% depending on the metropolitan region.

The relationship between the cost of the healthy basic food basket and the value of the minimum wage showed a downward trend between 2009 and 2015, interrupted by a sequence of increases in the amount needed to buy healthy food in 2016. From 2008, there was a change in the rules for adjusting the minimum wage, which would incorporate a percentage in relation to the real growth in gross domestic product over the previous two years. This led to a phase of appreciation of the minimum wage, simultaneously with the implementation of income transfer programs, to which many authors attribute the reduction in poverty and social inequality observed in Brazil over the same period^12^. From 2015 onwards, the beginning of the economic crisis in the country resulted in the installation of a political crisis, compromising the country’s growth as well as the appreciation of the minimum wage. In recent times, there have also been problems related to the Covid-19 pandemic, the war between Russia and Ukraine and the increasing impact of climate change in episodes of extreme events, with an influence on food production; this set of elements has contributed to the scenario identified in the study. The simultaneous occurrence of these scenarios has led to an increase in food prices globally, with a substantial rise in commodity prices in March 2022^13^. A similar trend is captured by the relationship between the cost of the basket and the average *per capita* income in the country. A period of relative stability was identified between 2012 and 2017, except for the increase observed in the economic and political context between 2016 and 2017, followed by an upward trend from 2020 onwards and a new period of stabilization from mid-2022 onwards. The information generated by defining the quantities and items in the healthy and sustainable basic food basket makes it possible to extract evidence for decision–making in public policies, beyond cost. Still on the economic side, it is possible to monitor the evolution of the share of prices of different food groups in the total cost of the basket. This allows for a continuous assessment of the items or food groups that have contributed substantially to variations in the cost of the basic food basket. The proposed basket can also be used as a reference for defining food programs and interventions, as a parameter for calculating monetary values in income transfer or food aid programs, as well as those involving the direct supply of food, such as the Workers’ Food Program, emergency actions and other actions and policies in the field of collective food. In addition, the increase in the price of fresh and minimally processed foods has been systematically higher than that of ultra–processed foods^14^. This trend may favor less healthy choices by the population, especially those on low incomes. Thus, the use and interpretation of the cost of the basket as an indicator of access to healthy food also benefits from monitoring the cost of and access to ultra-processed foods.

Some of the study’s methodological issues are worth highlighting. Firstly, the process of delimiting the food groups and their respective quantities for a healthy basic food basket, although based on evidence from scientific studies and public health recommendations, was based on criteria stipulated by the authors of the study. However, it is important to point out that recommendations proposed in recent scientific literature on the subject were selected, also considering elements of the characteristics of the places where the food basket proposals listed were implemented. The choice of Eat-Lancet as a reference diet was based on the robustness of the evidence selected by a team of researchers with expertise in the subject, as well as its grounding in the current literature on the effects of food on human and planetary health. Eat-Lancet, however, is a global reference, the quantities of which must be adapted to different local contexts. In this sense, although the total amount of red meat did not exceed the recommended limit (28 g), the amount of beef was substantially higher than the amount of pork – 24 g and 4 g, respectively (the reference diet proposes a maximum of 14 g for each type of red meat). There is no apparent impact on health, since the literature discusses the effect of total red meat on the risk of disease^15^. The environmental impacts are relatively small. A study that evaluated changes to the Brazilian diet estimated that an average population consumption of 45 g/day would be compatible with a reduction of approximately 50% in various environmental impact indicators, such as greenhouse gas emissions and particulate matter, land use, water use, eutrophication, among others^10^. Therefore, the proposed basket preserves its characteristic of being compatible with lower environmental impact. Due to the historical series of inflation indices for the metropolitan region of Vitória (from January 2014), it was only possible to update prices for this territory from January 2019. There are methodological differences between the POF price records and the monthly price surveys carried out by the SNIPC. Therefore, the estimated cost for the Vitória metropolitan region for January 2018 (estimated from the 2017–2018 POF) and the cost for January 2018 for the other metropolitan regions (estimated from the 2008–2009 POF and updated using inflation indices) are not directly comparable.

The selection and determination of the quantities of food in each group was based on food acquisition information from the 2008–2009 and 2017–2018 POFs, so foods with greater household availability were included in greater proportion in the proposed basic food baskets. The use of real data on food purchases by the population is based on the accessibility and acceptability of the items by the population, avoiding the incorporation of extreme changes in dietary patterns and considering the preservation of cultural aspects of food in the context of the proposed healthy basic food basket, both at a national level and in metropolitan regions.

In short, for both Brazil and the metropolitan regions, there was an increase in the real cost of the basic food basket, with a more substantial increase from 2018 onwards. The downward trend since 2009 in the ratio between the value of the basket and the value of the minimum wage was interrupted, with an upward trend from 2018 onwards. Compared to the average *per capita* income, the value of the basket fluctuated over the period, with an upward trend from 2018 onwards.
