# Supplementary material for: Cost of a healthy and sustainable basic food basket in Brazil between 2009 and 2024
Source: Rev Saude Publica. 2026 Apr 10;60:e15. doi: 10.11606/s1518-8787.2026060006878 (PMC13073137; doi:10.11606/s1518-8787.2026060006878)
Supplement: Translation

# Translation: Custo da cesta básica saudável e sustentável no Brasil entre 2009 e 2024

**Authors:** Eliseu Verly, Dirce Maria Lobo Marchioni, Semíramis Martins Álvares Domene, Isabela de Albuquerque Ribeiro, Flávia Mori Sarti

**Affiliations:** I Universidade do Estado do Rio de Janeiro. Instituto de Medicina Social. Departamento de Epidemiologia. Rio de Janeiro, RJ, Brasil; II Universidade de São Paulo. Faculdade de Saúde Pública. Departamento de Nutrição. São Paulo, SP, Brasil; III Universidade Federal de São Paulo. Instituto de Saúde e Sociedade. Departamento de Políticas Públicas e Saúde Coletiva. São Paulo, SP, Brasil; IV Universidade de São Paulo. Escola de Artes, Ciências e Humanidades. Gestão de Políticas Públicas. São Paulo, SP, Brasil

**Keywords:** Alimentação Saudável, Alimentação Básica, Índice de Preços ao Consumidor, Análise de Custos

## Abstract

**OBJETIVO:**

Avaliar a evolução do custo de uma proposta de cesta básica saudável e sustentável, nacional e para as regiões metropolitanas, entre 2009 e 2024.

**MÉTODOS:**

Foram utilizados dados de aquisição de alimentos (quantidades e valores pagos) provenientes das Pesquisas de Orçamentos Familiares de 2008–2009 e 2017–2018. Os grupos de alimentos que compõem as cestas foram baseados na dieta planetária *Eat-Lancet* e em instrumentos regulatórios oficiais*.* O Índice de Preço ao Consumidor Amplo referente aos alimentos e grupos de alimentos foi utilizado para atualização mensal dos preços dos itens consumidos pela população brasileira no contexto das Pesquisas de Orçamentos Familiares que compõem a cesta básica proposta. Descontou-se a inflação acumulada no período para comparação de valores monetários ao longo do tempo, calculando-se o valor real das cestas básicas.

**RESULTADOS:**

O custo real da cesta básica nacional proposta manteve-se relativamente estável entre 2009 e 2012, apresentando posterior tendência de crescimento até início de 2017, incluindo crescimento mais expressivo entre 2018 e 2024. Padrão similar foi observado nas cestas básicas das regiões metropolitanas. O custo (adulto/mês) representou em média 26% do salário-mínimo vigente em cada ano do período de análise e 19% da renda *per capita* média nacional entre 2010 e 2023. A participação dos grupos de alimentos no custo da cesta proposta manteve-se estável no período.

**CONCLUSÃO:**

Houve um aumento no custo real da cesta básica saudável e sustentável proposta em nível nacional e regional, particularmente a partir de 2018. Ademais, o valor da cesta básica saudável e sustentável apresentou tendência de aumento em relação à renda média *per capita* da população, especialmente a partir de 2018.

## INTRODUÇÃO

A primeira iniciativa de estabelecer uma cesta básica de alimentos no Brasil foi feita pelo decreto-lei que regulamentou o salário-mínimo (Decreto-Lei n° 399, de 1938), definida como remuneração mínima necessária para atendimento das necessidades diárias de “*alimentação, habitação, vestuário, higiene e transporte*” dos trabalhadores em determinada época e região do país. O cenário epidemiológico no país era centrado no problema da fome e carências nutricionais, particularmente em termos de comprometimento da produtividade da força de trabalho pelo adoecimento causado pela desnutrição. O custo da cesta básica de alimentos estipulada ao final da década de 1930 tem sido utilizado por décadas como indicador de custo de vida e acesso à alimentação básica para população, assim como parâmetro para estimativa do salário mínimo no país, embora o valor real da remuneração tenha apresentado defasagens em vários períodos da história^1^.

Recentemente, uma nova composição de cesta básica de alimentos foi definida por meio de decreto presidencial, visando subsidiar políticas e programas para garantia do direito humano à alimentação adequada. O decreto estabelece a observância às recomendações do Guia Alimentar para a População Brasileira como uma das diretrizes para a composição da cesta básica, sendo vedada a inclusão de alimentos ultraprocessados. A mudança na composição da cesta básica também busca incentivar a promoção de sistemas alimentares sustentáveis ao considerar o uso de recursos naturais e emissões de gases de efeito estufa envolvidos na produção e comercialização dos alimentos. A nova cesta básica inclui dez grupos de alimentos, majoritariamente formados por alimentos *in natura* ou alimentos minimamente processados. Outros aspectos do sistema alimentar, como incentivo a alimentos provenientes da sociobiodiversidade e aceitabilidade cultural, estão contemplados pelo decreto, devendo ser observados na proposição das cestas básicas, isto é, na definição dos tipos de alimentos de cada grupo e suas respectivas quantidades^2,3^.

A renda e o preço dos alimentos constituem barreiras para acesso e aquisição de alimentos de boa qualidade em quantidade suficiente para manutenção da saúde da população^4^. O papel dos determinantes sociais no processo alimentação-nutrição-saúde tem sido destacado na literatura em nível mundial, apontando a relação entre condições socioeconômicas e aquisição ou consumo de alimentos^5^. Estudos empíricos sistematicamente apontam incremento na demanda de alimentos saudáveis a partir do aumento da renda da população ou a partir da redução dos preços dos itens alimentares associados a padrões alimentares saudáveis^6,7^. Assim, o custo de uma cesta básica de alimentos saudáveis constitui indicador importante para monitoramento do acesso da população a um conjunto mínimo de condições de vida pautadas nas demandas nutricionais. Portanto, o objetivo do presente estudo foi avaliar a evolução do custo real e nominal de uma cesta básica saudável no Brasil e regiões metropolitanas nos últimos 15 anos, entre 2009 e 2024. Ademais, foi estimada a trajetória da proporção do orçamento familiar necessária para aquisição da cesta básica proposta no presente estudo, bem como a evolução da participação dos custos de cada grupo de alimentos em relação ao custo total da cesta básica de alimentos.

## MÉTODOS

### Fonte dos Dados

A base de dados empregada para seleção dos alimentos e suas respectivas quantidades foi extraída das informações de aquisição domiciliar de alimentos, coletadas no âmbito da Pesquisa de Orçamentos Familiares (POF) conduzida pelo Instituto Brasileiro de Geografia e Estatística (IBGE) nos anos de 2008 e 2009. Produtos alimentares adquiridos pelos membros do domicílio, durante o período de coleta dos dados, foram registrados na caderneta de aquisição coletiva no contexto da POF, incluindo: quantidade adquirida, local, forma de aquisição e valor gasto. Como a coleta dos dados ocorreu ao longo de 12 meses, os custos relatados para cada item de compra foram ajustados para um único período de referência (janeiro/2009) utilizando o Índice de Preços ao Consumidor Amplo (IPCA). Informações sobre amostragem, coleta dos dados e demais procedimentos metodológicos das POF podem ser obtidos no site da pesquisa^8^. Exclusivamente no caso da região metropolitana de Vitória foram utilizados dados referentes à POF 2017–2018 para coincidir com períodos em que há disponibilidade de índices de inflação para atualização dos valores monetários da região. Os procedimentos amostrais e de coleta de dados foram similares nas duas edições da POF.

### Definição da Composição da Cesta de Alimentos

Inicialmente, foram definidos os grupos de alimentos para composição das cestas básicas nacional e regionais. Como referenciais teóricos para construção da proposta de cesta básica, foram considerados documentos do governo federal referentes à Emenda Constitucional n^o^ 132 (de 20 de dezembro de 2023), ao Decreto n^o^ 11.936 (de 5 de março de 2024), e à Portaria do Ministério do Desenvolvimento Social e Combate à Fome n° 966 (de 6 de março de 2024). Estes documentos aderem aos princípios de uma alimentação saudável do Guia Alimentar para a População Brasileira, definindo os grupos de alimentos da cesta e vedando o uso de qualquer alimento ultraprocessado. A seguir, na definição das quantidades de cada um dos grupos de alimentos, o principal referencial teórico foi a *Eat-Lancet Reference Diet* (dieta planetária)^9^. Esta publicação traz uma faixa de valores recomendados de consumo para os grupos de alimentos dos quais há evidências suficientes na redução do risco de doenças crônicas não transmissíveis. Nesta etapa, algumas adaptações foram realizadas:

- Dada a importância cultural e nutricional das carnes vermelhas, foi definida a quantidade máxima proposta pela dieta planetária, o que equivale a aproximadamente duas porções de 100 g por semana (peso bruto de 40 g por dia, que equivale a 28 g após cocção). Optamos por seguir a proporção entre carne bovina e carne suína conforme reportado pela população brasileira, o que levou a uma quantidade de carne bovina acima do limite estabelecido pela dieta planetária. As implicações desta alteração são abordadas na seção de discussão.
- Foram definidas três porções de 100 g de frango e uma porção de 150 g de peixe por semana (peso bruto equivalente a 430 g e 215 g por semana, respectivamente).
- Considerando a aceitação cultural do feijão, bem como sua importância como fonte de nutrientes, foi definido um valor intermediário entre o consumo observado na população e o valor otimizado para adequação nutricional^10,11^.
- A quantidade de oleaginosas (como as castanhas) foi reduzida a 5 g por não ser um item frequentemente presente na dieta do brasileiro, além de ser de alto custo.
- Foram definidas duas porções ao dia de frutas e quatro porções de legumes e verduras, assim como uma porção de tubérculos e vegetais amiláceos.
- A quantidade de cereais, que inclui cereais integrais, beneficiados e derivados (pães e macarrão) foi calculada para completar o valor energético de 2.000 kcal, com o limite de 60% do total de calorias da cesta. Foi adotado 20 g para o arroz integral (equivalente a 50 g pós-cocção); esta quantidade pode ser alcançada com a substituição de 30% das ocasiões em que o arroz branco é consumido na população, com substancial impacto estimado nas mortes por doenças crônicas não transmissíveis^10^.
- O café foi incluído para representar o grupo “Café, chá, mate e especiarias” por ser o item de relato majoritário neste grupo. Como não há valor de recomendação para consumo de café, foi adotada na cesta proposta a mesma quantidade média de compra relatada por cada unidade de federação.

A Tabela apresenta a distribuição dos grupos de alimentos segundo a proposta da dieta planetária e a presente proposta de cesta de alimentos para o Brasil. A composição da cesta foi apresentada como quantidades dos alimentos a serem adquiridas, referida como “cesta aquisição” (pesos brutos) e quantidades a serem efetivamente consumidas, referida como “cesta consumo” (pesos corrigidos pela cocção e porções não comestíveis).

**Tabela:** Quantidades de referência e quantidades propostas dos grupos de alimentos para compor a cesta básica nacional (quantidades brutasa e ajustadasb), Brasil.

	Eat-Lancet reference diet^a^	Cesta (consumo^b^)	Cesta (aquisição^c^)
Cereais	≤ 60%^d^	37%^d^	37%^d^
Arroz integral		50	20
Arroz branco		225	90
Pães		50	50
Macarrão		23	10
Tubérculos e vegetais amiláceos	50 (0–100)	30	45
Hortaliças	300 (200–600)	200	250
Frutas	200 (100–300)	210	300
Laticínios			
Leite	250 (0–500)	150	150
Queijo		20	20
Manteiga		5	5
Carne vermelha	14 (0–28)		
Carne bovina		24	34
Carne suína		4	6
Aves	29 (0–58)	43	60
Peixes	28 (0–100)	21	30
Ovos	13 (0–25)	20	20
Leguminosas	50 (0–100)	200	80
Amendoim, nozes e castanhas	50 (25–75)	5	5
Óleos insaturados	40 (20–80)	35	35
Açúcar de adição	31 (0–31)	10	10
Café, chá, mate e especiarias^e^	-	6	6

^a^ Valor de referência da dieta e variação possível (gramas/dia).

^b^ Peso, em gramas, removendo partes não comestíveis e ajustando por fatores de cocção.

^c^ Peso bruto em gramas (quantidade a ser comprada). As quantidades de referência são referentes às quantidades efetivas de consumo.

^d^ Participação em relação ao total de calorias da cesta.

^e^ Não há recomendação de consumo para este item.

Optamos por incluir todos os alimentos relatados de cada grupo, uma vez que todos podem compor a cesta. As quantidades propostas foram proporcionais às referidas na compra (*per capita*) pelas famílias, e somadas até atingir as quantidades estabelecidas em cada grupo de alimentos. A título de exemplo, a média diária per capita de aquisição de banana no país foi de 16 g, o que representa 28% do total de frutas adquiridas (59 g). Na composição da cesta, a participação da banana permanece a mesma (28%), o que equivale a 84 g diante do total de frutas que deve compor a cesta (300 g). O cálculo, no entanto, foi feito para cada estado e Distrito Federal e a média nacional foi calculada como a média de suas quantidades entre estados, ponderada pelo tamanho populacional de cada estado. Este procedimento permitiu considerar a variabilidade de composição da cesta, conferindo mais peso aos itens alimentares que são consumidos com mais frequência, respeitando assim a regionalidade cultural da alimentação.

### Cesta de Alimentos por Região Metropolitana

Este procedimento foi repetido para cada uma das 10 regiões metropolitanas consideradas pelo IBGE para construção dos índices de preços. Em termos de delimitação amostral das regiões metropolitanas, foram agregados domicílios das unidades primárias de amostragem, em cada unidade de federação, pertencentes às capitais e regiões metropolitanas, conforme descrito na documentação da pesquisa. As regiões metropolitanas avaliadas são: Belém, Fortaleza, Recife, Salvador, Vitória, Belo Horizonte, Rio de Janeiro, São Paulo, Curitiba e Porto Alegre.

As quantidades *per capita* por dia dos grupos de alimentos para composição das cestas básicas de alimentos saudáveis propostas (Brasil e Regiões Metropolitanas) foram ajustadas para fornecerem aproximadamente 2.000 kcal/pessoa/dia.

### Custo Mensal dos Alimentos e das Cestas

As variações nos preços de cada alimento, mensurada pelo Sistema Nacional de Índice de Preços (SNIPC) por meio do IPCA, foram utilizadas para atualização do custo da cesta básica. Para a cesta brasileira, foi utilizado o IPCA estimado para o Brasil, e para as regiões metropolitanas, seus respectivos índices. Cada item de cada cesta básica foi pareado com seu respectivo item do SNIPC. Em caso de ausência do índice de preços de um determinado item em um determinado período, utilizou-se registro referente ao grupo de alimentos. O preço, por quilograma, de referência para cada alimento foi obtido do módulo de despesa coletiva da POF 2008–2009 (2017–2018 exclusivamente para região metropolitana de Vitória), cujo período de referência era janeiro de 2009 (janeiro de 2018, no caso da POF 2017–2018). A atualização monetária dos valores de cada item alimentar *i* componente das cestas básicas para os meses subsequentes (fevereiro de 2009 a dezembro de 2024, ou fevereiro de 2018 a dezembro de 2024 para a região metropolitana de Vitória) foi baseada na seguinte equação:


$$p_{i}^{t+1}=p_{i}^{t}\times \left(1+\pi_{i}^{t,t+1}/100\right)$$

Sendo: $p_{i}^{t+1}=\text{ preço do item }i$ no período *t+1*; $p_{i}^{t}=\text{ preço do item }i$ no período *t*; $\pi_{i}^{t,t+1}=\text{ variação no preço do item}i$ entre *t* e *t+1* (IPCA referente ao mês t+1 para cada item).

Assim, foi calculado o valor nominal, isto é, o valor efetivo referente a cada período, para cada item, e calculado o custo nominal total da cesta básica. Para fins de comparação ao longo do tempo, faz-se necessário descontar a inflação acumulada no período. A partir deste procedimento, chamado de deflacionamento, foram calculados os custos reais das cestas básicas no período. A seguinte equação foi utilizada:


$$vr_{t}=\frac{vn_{t}}{IPCA_{t0-t}/IPCA_{t0-ref}}$$

Sendo: *vr*_t_ = custo da cesta básica no mês *t* descontado o valor da inflação, *vn*_t_ = custo da cesta básica efetivamente praticado no mês *t*, e *IPCA*_t0-t_ e *IPCA*_t0-ref_ = variações acumuladas do índice geral de inflação (Brasil ou regiões metropolitanas) entre *t0* (janeiro de 2009 ou janeiro de 2018) e o mês *t*, e entre *t0* e o mês de referência (dezembro de 2024), respectivamente.

### Participação dos Grupos de Alimentos no Custo da Cesta

A cada mês da série histórica, calculou-se a participação de cada grupo de alimentos no custo global de cada cesta básica, buscando-se identificar o respectivo peso na determinação do custo de vida da população.

### Participação no Orçamento Familiar

A cada mês da série histórica, foi calculada a proporção da renda domiciliar *per capita* média empregada para aquisição da cesta básica de alimentos *per capita*. As estimativas de renda *per capita* média no Brasil foram obtidas a partir da Pesquisa Nacional por Amostragem de Domicílios contínua, cuja série histórica inicia-se em 2012. Adicionalmente, calculou-se a relação entre o custo mensal das cestas básicas de alimentos para um indivíduo adulto e o valor do salário-mínimo vigente no respectivo período (expressa em percentual). Para estes cálculos, foram utilizados os valores nominais de rendimento, salário-mínimo e custo das cestas.

## RESULTADOS

O custo *per capita* da cesta básica de alimentos saudável e sustentável no Brasil é apresentado na Figura 1. O valor nominal para aquisição dos alimentos parte de R$ 4,25 em janeiro de 2009 e atinge R$ 13,36 em dezembro de 2024. Em valores reais (corrigidos para dezembro de 2024), o custo seria R$ 10,39 em janeiro de 2009. O custo real se manteve relativamente estável até meados de 2012, com tendência de crescimento até início de 2017. Após um rápido período de redução, até início de 2018, houve uma tendência de aumento expressivo, que se manteve por todo o período subsequente. Em novembro de 2019, custo real da cesta básica era praticamente o mesmo de janeiro de 2018 (R$ 10,38 *versus* R$ 10,36). A partir deste ponto, houve um aumento acumulado de 28% até dezembro de 2024.

**Figura 1:** Custo real e nominal da cesta básica (adulto/dia) em reais. Brasil, 2009 a 2024.

A relação entre custo mensal da cesta básica saudável para um adulto e valor do salário-mínimo foi marcada por uma redução entre 2009 e 2017, atingindo o valor mínimo de 23% em dezembro de 2017. Tendência de aumento foi observada a partir de 2018, atingindo valor máximo de 30% em novembro de 2022 (Figura 2). A média no período avaliado foi de 26%. Já a relação entre o valor da cesta mensal por indivíduo adulto e a renda domiciliar *per capita* seguiu estável em torno de 18% até final de 2019, com tendência de aumento nos meses seguintes e aparente redução a partir do início de 2023 (Figura 3). Não houve mudança significativa na participação de cada grupo de alimentos na composição do custo total da cesta entre 2009 e 2024. Frutas e hortaliças ocuparam, em torno de 30% do total do custo; seguidas pelas carnes (17%), cereais (15%), laticínios (11%) e ingredientes (óleo, sal, açúcar, azeite), somando aproximadamente 10% (Figura 4).

**Figura 2:** Percentual do salário-mínimo necessário para aquisição da cesta básica saudável. Brasil, 2009 a 2024.

**Figura 3:** Percentual da renda *per capita* média necessário para aquisição da cesta básica saudável. Brasil, 2012 a 2023.

**Figura 4:** Participação relativa (%) dos custos de cada grupo de alimento em relação ao custo total da cesta. Brasil, 2009 a 2024.

A evolução do custo das cestas regionais seguiu mesmo padrão da cesta nacional, com trajetórias semelhantes entre as regiões metropolitanas. As cestas que se mantiveram de menor custo na maior parte do período avaliado foram referentes às regiões metropolitanas de Fortaleza, Belém e Belo Horizonte. Por outro lado, as cestas de maior custo foram, na maioria das vezes, referentes às regiões metropolitanas de São Paulo e Rio de Janeiro (Figura 5).

**Figura 5:** Custo real da cesta básica (adulto/dia) em reais. Regiões Metropolitanas, 2009 a 2024.

## DISCUSSÃO

No presente artigo, avaliamos a evolução do custo de uma proposta de cesta básica para o Brasil e regiões metropolitanas, bem com sua participação no orçamento familiar mensal. Embora o custo nominal seja relevante por representar a quantidade efetivamente paga em um determinado momento, sua interpretação é limitada, pois carrega o efeito distorcivo da inflação. A observação isolada de que os custos aumentam ao longo do tempo se torna insuficiente, uma vez que esta variação é acompanhada, tipicamente, por ajustes nos rendimentos monetários da população. Por outro lado, a análise do custo real remove o efeito da inflação, permitindo identificar variações na capacidade de compra. Assim, a partir dos custos reais, para aquisição dos itens incluídos nas propostas de cestas de alimentos saudáveis, é possível identificar um período longo de estabilidade (janeiro de 2009 a setembro de 2018) seguido por um aumento acentuado, acumulando 29% no custo da cesta proposta ao longo do período investigado. Este incremento variou de 15% a 30% de acordo com a região metropolitana.

A relação entre o custo da cesta básica de alimentos saudáveis e o valor do salário-mínimo apresentou trajetória de queda entre 2009 e 2015, interrompida por uma sequência de aumentos no valor necessário para aquisição dos alimentos saudáveis em 2016. A partir de 2008, houve uma mudança nas regras de reajuste de salário-mínimo, que incorporaria um percentual em relação ao crescimento real do produto interno bruto nos dois anos anteriores. Isto levou a uma fase de valorização do salário mínimo, simultaneamente à implementação de programas de transferência de renda, aos quais muitos autores atribuem a redução da pobreza e desigualdade social observada no Brasil no mesmo período^12^. A partir de 2015, o início de crise econômica no país resultou em instalação de uma crise política, comprometendo o crescimento do país bem como a valorização do salário-mínimo. Em tempos recentes, somaram-se problemas relacionados à pandemia de covid-19, à guerra entre Rússia e Ucrânia e ao impacto cada vez maior de mudanças climáticas em episódios de eventos extremos, com influência na produção de alimentos; este conjunto de elementos contribuiu para o cenário identificado no estudo. A ocorrência simultânea de tais cenários tem gerado aumento nos preços dos alimentos em nível global, observando-se substancial elevação nos preços das *commodities* em março de 2022^13^. Tendência semelhante é capturada pela relação entre o custo da cesta e a renda média *per capita* no país. Identificou-se período de relativa estabilidade entre 2012 e 2017, à exceção do aumento observado no contexto econômico e político entre 2016 e 2017, seguido de tendência de aumento a partir de 2020 e novo período de estabilização a partir de meados de 2022. As informações geradas a partir da definição das quantidades e dos itens da cesta básica de alimentos saudável e sustentável permitem extrair evidências para tomada de decisão em políticas públicas, para além do custo. Ainda no aspecto econômico, é possível monitorar a evolução da participação dos preços de diferentes grupos de alimentos no custo total da cesta. Isto permite uma avaliação contínua dos itens ou grupos de alimentos que têm contribuído de forma substancial para variações no custo da cesta básica. A proposta de cesta também poderá ser usada como referência para definição de programas e intervenções em alimentação, como parâmetro para o cálculo de valores monetários em programas de transferência de renda ou auxílios-alimentação, como aqueles que envolvem a oferta direta de alimentos, como o Programa de Alimentação do Trabalhador, ações emergenciais e outras ações e políticas no campo da alimentação coletiva. Além disso, o aumento nos preços dos alimentos *in natura* e minimamente processados tem sido sistematicamente maior que nos alimentos ultraprocessados^14^. Esta tendência pode favorecer escolhas menos saudáveis pela população, especialmente de baixa renda. Assim, o uso e interpretação do custo da cesta como um indicador de acesso a uma alimentação saudável se beneficiam também do monitoramento do custo e acesso aos alimentos ultraprocessados.

Algumas questões metodológicas do estudo merecem destaque. Primeiro, o processo de delimitação dos grupos de alimentos e respectivas quantidades para composição de uma cesta básica de alimentos saudáveis, ainda que fundamentado em evidências provenientes de estudos científicos e recomendações em saúde pública, foi baseado em critérios estipulados pelos autores do trabalho. Entretanto, é importante apontar que foram selecionadas recomendações propostas na literatura científica recente no tema, considerando também elementos das características dos locais de implementação das propostas de cestas básicas elencadas. A escolha da *Eat-Lancet* como uma dieta de referência foi baseada na robustez das evidências selecionadas por equipe de pesquisadores com expertise no assunto, assim como em vista de sua fundamentação na literatura atual sobre efeitos da alimentação na saúde humana e planetária. A *Eat-Lancet*, no entanto, é uma referência global, cujas quantidades devem ser adaptadas para diferentes contextos locais. Neste sentido, ainda que o total de carnes vermelhas não tenha ultrapassado o limite recomendado (28 g), a quantidade de carne bovina foi substancialmente maior que a quantidade de carne suína – 24 g e 4 g, respectivamente (a dieta de referência propõe no máximo 14 g para cada tipo de carne vermelha). Não há aparente impacto na saúde, uma vez que a literatura versa sobre o efeito das carnes vermelhas totais no risco de doenças^15^. Os impactos ambientais são relativamente pequenos. Um estudo que avaliou modificações na dieta brasileira estimou que um consumo médio na população de 45 g/dia seria compatível com uma redução de aproximadamente 50% em diversos indicadores de impactos ambientais, tais como emissão de gases de efeito estufa e material particulado, uso da terra, uso da água, eutrofização, entre outros^10^. Logo, a cesta proposta preserva sua característica de ser compatível com menor impacto ambiental. Em função da série histórica de índices de inflação para região metropolitana de Vitória (a partir de janeiro de 2014), foi possível atualizar os preços para este território somente a partir de janeiro de 2019. Há diferenças metodológicas entre o registro de preço das POF e as tomadas mensais de preço pelo SNIPC. Assim, o custo estimado para a região metropolitana de Vitória referente a janeiro de 2018 (estimado a partir da POF 2017–2018) e o custo referente a janeiro de 2018 para as demais regiões metropolitanas (estimados a partir da POF 2008–2009 e atualizados utilizando os índices de inflação) não são diretamente comparáveis.

A seleção e a determinação das quantidades dos alimentos em cada grupo foram baseadas nas informações de aquisição de alimentos da POF 2008–2009 e 2017–2018, assim, alimentos com maior disponibilidade domiciliar foram incluídos em maior proporção nas cestas básicas de alimentos propostas. O uso de dados reais de aquisição de alimentos pela população é fundamentado na acessibilidade e aceitabilidade dos itens pela população, evitando incorporação de mudanças extremas no padrão alimentar e contemplando a preservação de aspectos culturais da alimentação no contexto da proposta de cesta básica de alimentos saudáveis, tanto em nível nacional, como nas regiões metropolitanas.

Em suma, tanto para o Brasil quanto para as regiões metropolitanas, registrou-se aumento no custo real da cesta básica, com incremento mais substancial a partir de 2018. A tendência de queda desde 2009 na relação entre o valor da cesta e o valor do salário-mínimo foi interrompida, com tendência de aumento a partir de 2018. Em comparação com a renda média *per capita*, o valor da cesta oscilou no período, com tendência de aumento também a partir de 2018.
